# Understanding adaptability in the family environment in facing COVID-19: A review

**DOI:** 10.1016/j.heliyon.2023.e20618

**Published:** 2023-10-27

**Authors:** Sitti Nursetiawati, Jenny Sista Siregar, Dian Pertiwi Josua

**Affiliations:** a Jakarta State University, Environmental Management Postgraduate Program, Indonesia; b Jakarta State University, Faculty of Engineering, Family Welfare Cluster, Cosmetology Education Study Program, Indonesia

**Keywords:** Adaptation to new habits, COVID-19, Family ecology, Family environment

## Abstract

Adaptation is a research field that is trending in the face of the coronavirus disease 2019 (COVID-19) outbreak in various parts of the country. The ability to adapt is one way for individuals to survive in uncertain situations. This article reviews the adaptation process in a family environment focusing on finding models from various literatures on family institution adaptations and then mapping them into adaptations that families can implement during the COVID-19 pandemic. Our study showed that family resilience in dealing with changes in various areas of life was performed through family adaptations during the COVID-19 pandemic, such as: (a) the ability of family members to be more open and respect privacy when communicating during all activities at home, (b) culture and values applied in the family are the determining factors for individuals to be able to adapt to new habits, (c) the resources owned by the family determine the attitudes and ways in which the family develops its potential in dealing with limitations and negative emotions, (d) the adaptive power of men as husbands or fathers with women as wives or mothers is driven by different factors, where men are driven generally due to external factors, while women due to internal factors. Families with different environments produce different adaptability, depending on the social capital and support received by the family.

## 1 Background

Coronavirus disease 2019 (COVID-19) has prompted major transformations in human life, and those who survive can adapt to face pressure situations (Meyer et al., 2022). COVID-19, the adaptation process is first familiarized with family institutions because individuals have lived with their families more often since the spread of the virus. The impact of COVID-19 environmentally different according to the zoning of distribution, namely the green zone for areas with a small risk of transmission, the yellow zone for areas with a small possibility of transmission still possible transmission, the orange zone is included as a zone with a relatively large number of transmissions, the red zone is the highest risk area, and the black zone is a category of high transmission and needs immediate treatment because many lives are threatened.

The capital city of Jakarta showed the highest spread of COVID-19 in Indonesia. Based on historical data on the distribution of COVID-19 in Indonesia, DKI Jakarta declared as many as 482,264 positive cases, with almost a quarter of the population (23.9 %) living in infected [1]. The uncontrollable coronavirus, which attacks quickly, has changed behavior of the people. Initially, people would freely carry out their activities outside the home, which has now become inversely proportional, where more than 720 (38.75 %) of Indonesian people admit that they leave their homes less often, and almost a quarter (24.63 %) admit that they only go out to do urgent work/activities during COVID-19 [2].

Economic and educational activities were conducted online. Previous studies have shown that the habituation of new adaptations in the world of effective education involves the family environment, both physically and non-physically, for a maximum learning process [3]. Individuals cannot adapt if they do not receive education and are formed directly by their family. Adaptation in the face of COVID-19 should be a form of interaction and institutionalization of new patterns of living habits that start with the family.

The crisis period has internal dynamics that are not only rational with procedures but also with traditional arguments, such that each individual when facing a crisis period can play a relevant role [4]. The COVID-19 crisis affects three strategies for adapting to short and long-term crises such that it affects changes in environmental conditions quickly [5]. COVID-19 is said to harm human privacy with respect to digital activities. Additionally, it is positively influenced by attitudes, norm subjectivity, and self-efficacy in maintaining privacy [6]. According to “Action” theory, the COVID-19 pandemic has affected human determinants and mechanisms in verifying their role [7].

Differences in the zoning of COVID-19 transmission result in fast or slow adaptation by the community. Typically, the success rate of adaptation, apart from zoning, is also in line with the habituation internalized by the family. When families can adopt new habits in the face of COVID-19, individuals also apply strict health protocols in the community because all habits always start at the family level. This research was conducted to map how adaptation develops in the family and connect it as a stimulus for adapting to new habits during COVID-19. Currently, research on adaptation has not discussed the forms and models derived from adaptation theories within the family unit according to COVID-19 conditions.

Previous studies have only discussed the form and level of community compliance by adapting new habits to reduce the spread of corona virus (Clark et al., 2020). The novelty of this research compared to that of the others is that it is a descriptive search for theories from literature and presents information formed as adaptation models to be applied during COVID-19. The unit of analysis referred to in this study was adaptation in the family environment, which was analyzed based on differences in gender, profession, and area of residence. COVID-19 has prompted major transformations in human life, and those who survive can adapt to the pressure of a situation. During COVID-19, the adaptation process first familiarized with family institutions because individuals have lived with their families more often since the spread of coronavirus.

## 2 Research methodology

This study us qualitative research with a systematic descriptive literature review obtained from the exploration of data provided by previous researchers, which is useful for readers to gain knowledge on the topic. Systematic literature reviews already exist in research and were widely used to review what was originally unstructured, systematic, and did not have transparency; therefore, it tends to be subjective to data and scientific interpretation. Literature was mostly obtained online from EBSCO, Scopus, and ABI/Inform, which can help researchers conduct systematic literature review research openly, and the results can be verified by the readers (Kraus, Breier, & Dasí-Rodríguez, 2020).

**Fig. 1:**
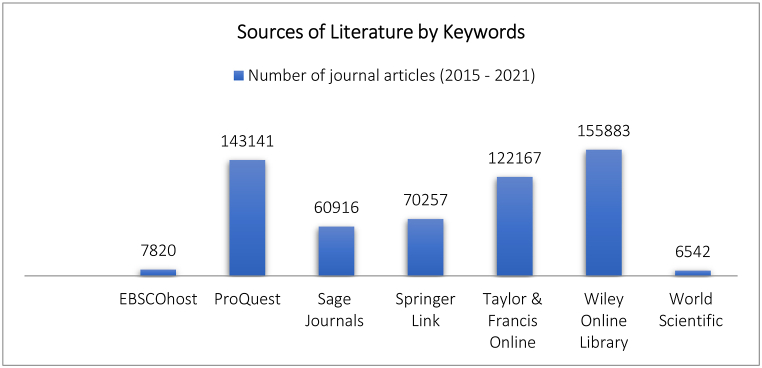
Bar plot depicting the systematic search sites used for the literature review. Source: Research data analysis (2021).

The literature study was conducted based on research on the theme of “Family Adaptation” using an online system originating from the online journal search sites (See Fig. 1) EBSCOhost (7820 articles), ProQuest (143,141 articles), Sage Journals (60,916 articles), Springer Link (70,257 articles), Taylor & Francis Online (122,167 articles), Wiley Online Library (155,883 articles), and World Scientific (6542 articles). Simultaneously, to observe the adaptation conditions of the families during COVID-19. Details were collected from scientific reports, Laws/Government Regulations, survey data in the form of news and newspapers obtained online, and reports on the incidence of COVID-19 in Indonesia. The objective of this study was limited to reviewing the model of adaptability in the Indonesian family environment based on literature dealing with COVID-19.

### 2.1 Generation of keywords

We searched for journal articles using the keyword “Family Adaptation” considering the novelty of the research, and selected scientific articles in the last 5 years from 2015 to 2021. Looking only at the relevant keywords, the total number of journals was 566, 726.

### 2.2 Journal search online

Considering the conditions that encourage the digitization of literature resources, we obtained journal articles from search engines that are often used through the accounts of the researchers by logging into https://e-resources.perpusnas.go.id/. The researchers chose several searches, including EBSCOhost, ProQuest, Sage Journals, Springer Link, Taylor & Francis Online, Wiley Online Library, and World Scientific. Then, we revisited each journal using the appropriate keywords. Additionally to avoid the research from expanding, we defined adaptation based on social, humanities, and psychosocial sciences.

### 2.3 Collection of relevant scientific journals

**Fig. 2:**
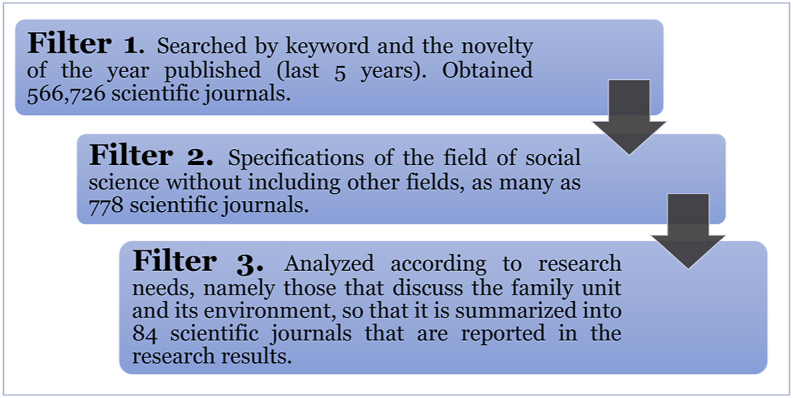
- Flow chart representing the process of filtering literature review source data. Source: Research data analysis (2021).

The scientific journals obtained were then analyzed, although only a few them were used in the study (Fig. 2). We examined adaptation in the family environment deeply by distinguishing gender, profession, and location according to the research criteria, namely, in the family unit. The differentiator examined differences in family adaptation to (a) father or husband, mother or wife, (b) type of work, and (c) area of residence in the form of family in rural and urban areas.

### 2.4 Description of the journal

The literature review was then processed and described as follows: (a) the definition of adaptation, (b) indicators of the family to adapt to the environment, and (c) any stimulus that encourages families to adapt to their environment. All review articles were reported in the form of tables, graphs, or descriptive studies.

### 2.5 Interpretation of findings

**Fig. 3:**
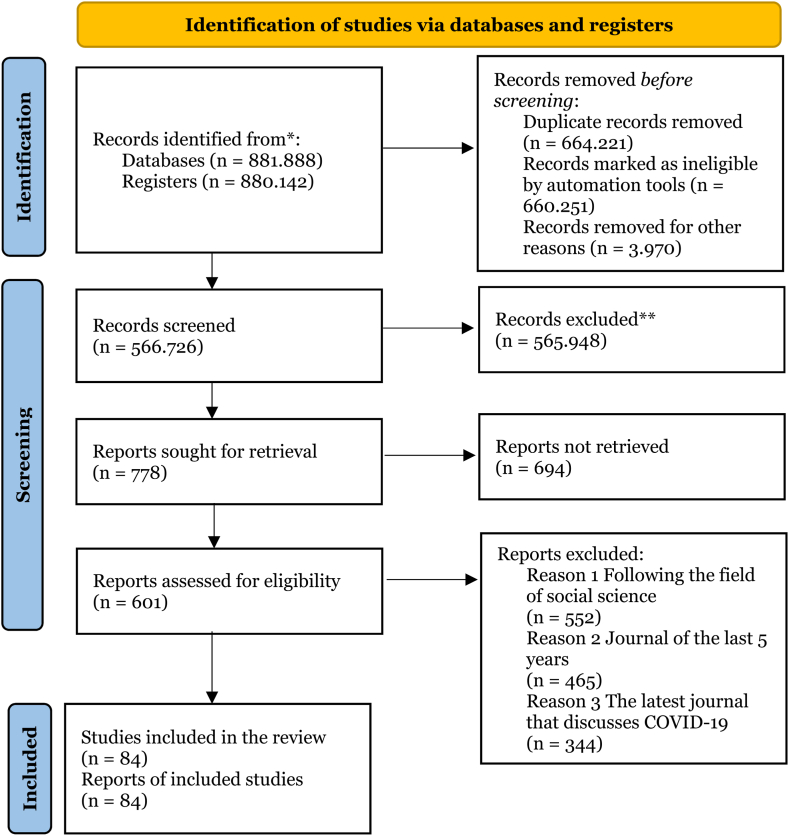
- PRISMA diagram depicting the process of the systematic literature review. Source: Research data analysis (2021) * If feasible, we reported the number of records identified from each database or register searched (rather than the total number across all databases/registers). **If automation tools were used, we indicated how many records were excluded by a human and how many were excluded by automation tools.

Based on the findings obtained in the literature review, we describe a model for adapting to the family environment during the COVID-19 pandemic. These models are based on both qualitative and quantitative literature studies emerged from theories and research findings related to adaptation to the family environment when facing life difficulties with a variety of triggers. The detailed process of the systematic literature review is described in the PRISMA diagram (Fig. 3) [8].

## 3 Results

The topic of adaptation was discussed in 206,117 journal articles. In this study, the topic was adapted to the research objective, namely, to see adaptation in the family environment. 67,377 journal articles appeared in the search for scientific journals. We identified 23 articles that were suitable for further research. In the family adaptation variable, we generally looked for a family coping with challenges that trigger stress, which we re-examined in a systematic literature review of 10 journal articles. Globally, families are trying to deal with epidemics and disasters, which result in uncertainty in life. Families require adaptation to survive negative impacts. For more than a year, COVID-19 has become an issue that has resulted in instability of life. Several changes have occurred a rapid pace, and humans must adjust to these changes quickly.

### 3.1 Basic theory of adaptation in the family environment

Ecological, sociocultural, psychosocial, and systems theories [9] are some of the foundations and grand theories of family adaptation (Table 1). The family is a microsystem with intrapersonal factors, one of which is demographic factors (age, education level, balance, life satisfaction, job satisfaction, and work-family conflict), which is a form of adaptive behavior [10,[11], [12]] to achieve a new balance between the needs of each family member to the integrity of the needs of the family as a whole when facing a crisis [13].

**Table 1:** Family adaptation system. By researcher (2021).

Family Adaptation System		Life Cycle	Aim	Process
1.	Parenting pattern	Children to adults	Parenting shapes the adaptability of children, so that when children can adapt, it will be easy to accept adaptation in the adult phase.	Make rules, form bonds, and build relationships between parents and children
2.	Environmental change	Long-term and short-term in individual	Drive change to occur within the individual strongly based on experience and environmental changes.	Anticipate and train someone to have adaptation strategies, discover potential, and develop their resources.
3.	Coping	When one of the family members is sick, separated from the nuclear family and social environment, and experiencing changes in routine	Adjusting to differences, confusion, and difficulties, reducing negative prejudices, the ability to face obstacles, and giving affirmation that individuals as family members who are going through a difficult phase of life still have strength and help from other family members	Opening positive communication, creating interactions that can reduce anger and despair, as well as family transactions with the social environment
4.	Family welfare	Availability of resources, conditions in which family members feel satisfied, fulfilled, happy, and have gratitude	Adapting to life situations	Understanding risks and dangers, instilling mutual respect among family members, receiving and responding to love and affection, respecting human nature, and exchanging needs for mutual fulfillment
5.	Emotional Response and regulation	Children to teenagers	Responses were given by family members, understanding of others, developing the capacity to understand and accommodate the needs and feelings of others	Accurate description of expressions and response cues, communication patterns as an adaptation style, and individuals as family members can be accepted by their environment and accept their environment
6.	Acculturation, assimilation, cultural processes, and family life history	Human life	Live life wisely, and understand that the essence of life is to change	Marrying different ethnic groups, having life events beyond human control, changing places of residence, getting new cultures and habits, meeting new people, and being part of a society with certain customs.

Parenting theory explains that fluctuations in family life, increased parental control and support for adolescents, problems with externalizing and internalizing values, and moderate adaptation in adolescents are not the primary factors that predict the ability of the adolescent to adapt [14] because they are getting older. At the age of the child, the behavioral control provided by the family decreases, and parenting practices begin to change with the association of adolescents with their external environment.

Family life cycle theory sparks psychological and social adaptation in a woman starting when a woman marries and raises children, and there is a close relationship between a adaptability of the woman to the family environment and her happiness when performing the roles of wife and mother [15]. Wives and mothers are believed to have a complex level of adaptation, especially when they must double as career women [10].

Ecological studies, state that the paradigm of adaptation to the potential, which focuses on environmental vulnerability, affects the way individuals face problems in everyday life related to human health and well-being [16]. Attachment theory, which interprets relational theory with respect to the adaptability of humans to challenges of life, states that emotion regulation is also influential at the beginning of human adaptation to the environment. The process is such that when humans know their inner world, they will understand and process their emotions, and when parents provide effective responses, children will internalize adaptive abilities and develop their capacity to understand their personal needs and feelings as well as the needs and feelings of others [17].

The processing of emotions as a picture of expression and accurate response cues produces a pattern of communication as an adaptation style of the person. When the individual feels accepted by his/her environment, or the individual accepts his environment, he/she will feel more secure, minimally hiding his/her own emotions, so that the adaptation becomes a signal of positive interaction and can respond to reciprocal relationships as good interpersonal relationships and reduce the negative effects of barriers to adaptation.

The ecological theory of human development commonly known as the family ecology theory initiated by Bronfenbrenner, conceptualizes the process of human adaptation as having the following levels: (a) at the onto system level related to parents, (b) a microsystem that is a family living space, (c) a mesosystem in the form of an external family that can help families adapt, (d) an ecosystem, namely community organizations in the health, education, and social sectors (social services), and (e) macrosystems in the development of socio-cultural attitudes towards the adaptation needs of family members who require adaptation [18].

The theory of family resilience, which was researched by Walsh in 1998 was later redeveloped by a subsequent study stating that the main component in adaptation is family resilience, which comprises the following four elements: (a) Family belief in looking at crisis situations this study measured through the concept of Family Sense of Control Index and found the meaning of difficulty and family strength as a way of looking at difficulties positively. The results also stated that family control is needed for adaptation through the ability to understand, self and emotional management, meaningfulness, and family harmony. (b) Organizational pattern, which refers to the integration of ownership of external and internal resources, as measured by stability of family roles, coherence, connectivity of family members, and balancing the interactions and roles of each family member. (c) The communication process measured using the Family Assessment Device to see that in adapting within the family using communication, emotional reactions, and problem solving skills. (d) Social support, which shows that in adaptation, families need to be open, should accept change, have mutual respect between family members, and emotional stability [19].

**Fig. 4:**
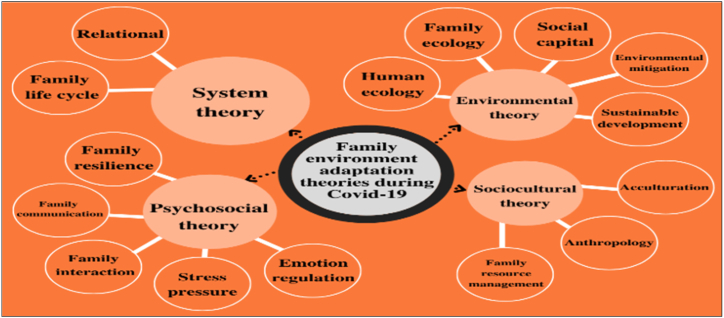
- Diagram illustrating the theories of adaptation to the family environment during COVID-19. Source: Research data analysis (2021).

The theoretical framework (See Fig. 4) of adjustment and adaptation proposed by McCubbin, Thompson, and McCubbin (1996) concludes that families that have experienced various life demands and limited financial and social support are more likely to have difficulty adapting. Contrarily, families who experience the same life demands but have a positive attitude and good problem-solving strategies, followed by strong family and community support, will be successful in adapting [20].

### 3.2 Family adaptation terminology

Recently, research on adaptation trending as COVID-19 cases have become a global epidemic. Adaptation is an interesting issue because it is an action that produces two forms: positively or negatively affecting non-market factors, such as water quality, ecosystem function, human health, social organization, and cultural practices [21]. Family is a determining factor in the ability of an individual to adapt (Table 2). Previous findings mention that childhood, in which individuals are still attached to their families and behave according to the wishes and formations of their families, integrate with the adaptation of someone at a later age and predict human adaptability [22].

**Table 2:** Family adaptation terminology. Based on research analysis (2021).

Provision	Reference
a. Family function quality b. Family resource management c. Family size	Mendes et al., 2016 [23]; Stefanos et al., 2020; Pickard & Ingersoll, 2017 [24]; Stochitoiu & Vadeboncoeur, 2020 [25]; Gardiner, Mâsse, & Larocci, 2019 [=[[26], [27], [28], [29]]
a. Family stress level b. Family life c. Family owned resources d. Family dynamics	Frishman et al., 2017 [30]; Guyard et al., 2017 [31]; Kahana et al., 2015 [[32], [33], [34], [35]]
a. Family demands b. Meaning of family	Jenaro et al., 2020 [36]; Fonseca et al., 2018 [37]; McStay, Trembath, & Dissanayake, 2015 [[38], [39], [40], [41]]
a. Moral principles b. Faith in goodness c. Interdependence and exchanging needs d. Cognitive regulation	Bazzan et al., 2019 [[42], [43]]
a. Human ability to understand the opportunity b. Ability to face challenges	Hatfield et al., 2018 [[44], [45]]

The drive for adaptation arises from a changing environment and experiences that make a person feel depressed, and family is one of the determinants of adaptation in a person. Families experience the effects of anticipation, adaptation, and strategies [46], which encourage these changes to last long or short term. However, family cannot completely change a person unless the individual wants a change in them strongly (Bricard, Legleye, and Khlat, 2017). Uncertainty, anxiety, and stress play a role in family adaptation and coping [[47], [48]].

Previous studies have stated that adaptation triggered by the environment, with the characteristics of family members being sick, separated from their nuclear family, friends, and social environment experiencing changes in daily routines interacting with and experiencing disturbances, such as negative mental health [49].

Adaptation based on other findings is encouraged because of the following factors: (a) cultural differences, (b) confusion when someone wants to feel happiness but has not yet achieved it, (c) economic difficulties, (d) social prejudice and feeling of isolation, (e) certain restrictions, (f) facing obstacles, (g) presence of others who provide strength and support, (h) acceptance of differences and motivation for a better future, (i) desire to grow together with the family, and (j) adaptation deliberately made by every human being to survive or maintain himself (Park, Kim, and Chung, 2021).

Adaptation can take the form of adjustments to social, sociocultural, psychological, psychosocial, ethnological, legal, economic, and ethnocultural aspects. The diversity of adaptation types in scientific methodologies can be studied through the realms of ethnopsychology, ethnoecology, ethnology, cultural anthropology, and the environment. In migrant families, adaptation is postulated as an ethnocultural adaptation involving the psychological, psychosocial, and social aspects of individuals to a new culture, traditions, customs, values, norms, attitudes, beliefs, rules of behavior, and way of life in the community that is in harmony so as not to cause conflict. The assimilation of a new culture can create inspiration, open communication, and build positive interactions that can avoid outbursts of anger and despair [50,51].

The family process creates positive adaptations in dealing with major crises, transitions that are quite disruptive to life, and other conditions that result in feelings of chronic stress in harmony with cultural diversity and family structure, potential resources, sociocultural influences, and developments of the times, as well as survival. This requires every member of the family to remain resilient. This process can also be considered a form of family transaction within the social environment. Positive adaptation also contextualizes the adaptation framework that is closely related to family resilience in the form of a belief system: a) the process of interpreting a certain issue; b) positive outlook, which is full of hope and affection among family members; c) transcendent values and spirituality that inspire, transform, and encourage each family member to grow and develop together both psychologically and in their physical health [52,53].

The family system states that adaptation to family members is generally conducted because of internal and external influences and problems that affect the subsystem of family functions. Adaptation indicates the quality of family functioning, which is closely related to management and family size. Each family has different variations in adaptation. Based on this study, health problems is a factor that causes families to adapt; for example, children suffer from chronic pain; therefore, not only one family member adapts but all family members need to adapt to the situation (Mastrotheodoros et al., 2020; Stochitoiu & Vadeboncoeur, 2020[25]; Gardiner et al., 2019[27]; Pickard & Ingersoll, 2017[24]; Mendes et al., 2016[23]).

Family adaptation is reflected in the level of family stress, family life, resources owned by the family, and factors that encourage families to adapt (stressors). Conclusively, family adaptation is not only in the form of physical conditions but also in the psychological state of each family member at risk within the family dynamics system [[30], [31], [32]].

**Fig. 5:**
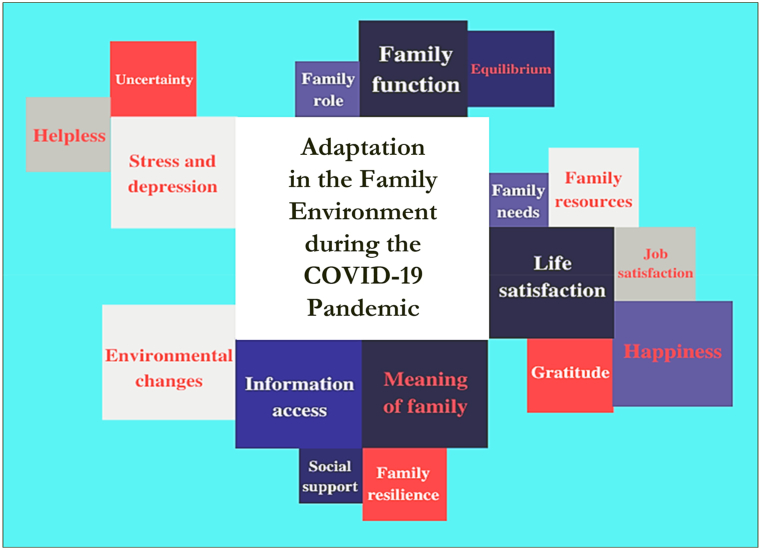
Chart showcasing the understanding of adaptation in the family environment in the COVID-19 pandemic situation. Source: Research data analysis (2021).

Adaptation mechanisms is related to family support (Fig. 5). Family members who share mutual support are happy in married life (Woods, Danes, and Uhalt, 2019). Adaptive coping of an individual can work well when the family cares, has unconditional love, is warm, and provides emotional support to family members. Families that support each other encourage high adaptability in their members [54]. Both events and experiences in every human life journey affect the decisions of family members to adapt and synchronize life plans [55] to reorganize their lives in the future. The social and structural functioning of the family from the family environment can be a reference for each family member to self-regulate and encourage successful adaptation to change, the ability to deal with conflict, understanding community norms to respect boundaries, rules, values, and principles (Galán-González, Martínez-Pérez, and Gascón-Catalán, 2021).

Family stress and measurable quality of family life are impacts of family dynamics; therefore, families need to determine whether adaptation will be successful or vice versa. In previous research, family adaptation can be observed using the Family Adjustment and Adaptation Response model by Patterson, which states that the initial concept of family adaptation lies in balancing demands and abilities through interaction; thus, it can be concluded that family adaptation according to this model is: (a) family demands in the form of normative and non-normative stressors, tensions, and the daily busyness of each family member (b) family capabilities are the resources owned by the family, psychosocial welfare, and family coping behavior, (c) the meaning of family is the family's assessment of the demands that require the family to adapt, cohesion, and the external environment, and (d) the result of the adaptation itself (Jenaro et al., 2020; Fonseca et al., 2018; McStay et al., 2015). This theory also states that adaptation is determined by the magnitude of the demands, meaning, and ability of family members to create new approaches to achieve balance [56].

**Fig. 6:**
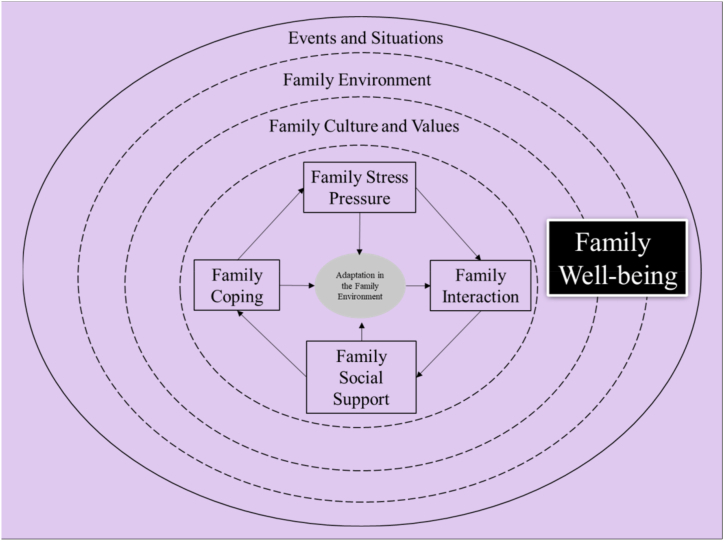
- Schematic of the family environment adaptation model. Source: Research data analysis (2021).

Adaptation systems involve the desire and ability to love, respect, and value others. Humans who can adapt will accept and respond to love, respect, and values given by others in a balanced way because they have understood that human nature is interdependent, giving each other a sense of comfort, exchanging needs so that they are fulfilled physically and mentally, and are confident (Fig. 6).

Adaptation is considered a cognitive regulation in which there is a stimulus, response, and acceptance. The result of adaptation is mutual support, which includes sharing the same space, experience, and suffering for humans to recover and gain the confidence to overcome obstacles [57]. Adaptation is a holistic system whose process begins with the activation of human cognitive and regulatory mechanisms through stimulation to defend oneself. The process takes the form of input (individual behavior itself, responses of other people to changes that occur in themselves, and their behavior, feedback, and coping) and output (environment) [42].

Eventually, adaptation is divided into four types: (a) positive adaptation, (b) passive adaptation with some preparations made to adapt, (c) adaptation due to frustration, and (d) adaptation due to the involvement of many factors, both internally and externally. All these four types of adaptations are experienced while adapting (Jeon, Ahn, and An, 2019). Additionally, three subcategories facilitate adaptation: (a) social support, (b) family abilities, and (c) spiritual beliefs and family religiosity. However four subcategories hinder family adaptation: (a) families or family members who require adaptation to limit themselves; (b) treatment conditions that do not support adaptation, such as excessive worry and fear of family members facing change [58]; (c) mental pressure caused by external parties; and (d) the urgency of traits or adaptation factors [59].

Acculturation is a process of fusion of culture with new cultural results, and in the family environment, it can also be referred to as a sustainable psychosocial adaptation process, which is an impact of the adapting individuals. Acculturation is a process of managing the lives of the family members in a new cultural context [60]. Thus, it exists because family members are able to adapt to new cultures without leaving the old culture as a whole, or by replacing the culture with habits that are more appropriate to the conditions and the environment. Another adaptation result is cognitive evaluation as a mediator of the determinants of the quality of life of a person [61].

Family adaptation is a product of the collective needs of family members to respond effectively to external disturbances and is a determinant of family resilience [62]. Maladaptation results in the deterioration of the emotional system of the family and every family member is cut off from other subsystems, resulting in fear, loss of control, inability of the family to grow and develop together, family chaos, excessive control systems, low self-esteem, resentment, and family members becoming private and grumpy.

Failure of family adaptation provides a bad experience for each family member, with the instillation of inappropriate values and behaviors. For example, when a father fails to adapt and is unable to carry out his responsibilities, relinquishes his role to the mother. The mother then takes full authority by becoming a single mother, which would disrupt and limit childcare, and a possibility of economic fulfillment not being accommodated arises (Henry, Sheffield Morris, and Harrist, 2015). Children will experience a decrease in academic achievement, emotional, social, physical, and spiritual disturbances due to anxiety, exposure to allergens or psychosomatic illnesses, and would be critical in relationships. They would also be at a risk of taking actions that can endanger themselves and others.

Adaptation is biologically correlated with anthropology and is intended as an aspect of the phenotype within an individual used to manage the risks posed by the environment (Hazel et al., 2021). Natural selection affects the family environment and encourages family institutions to adapt to risks. In social sciences, adaptation is a substance of social capital [63,64]. Adaptation does not occur only because individuals want to adopt or are required to make adjustments to their community and family environment, but also because of transmission through genetics, epigenetics, and culture. Thus, adaptation can be said to lead to the population level.

Family adaptation refers to a group of individuals who build a household through marriage, and it has implications for evolutionary demography. Although future environmental conditions are most likely a reflection of the past conditions of an individual, adaptation can change history and encourage humans to change into individuals who are prepared to face future changes [[65], [66], [67]].

The process of family adaptation begins not only when a man marries a woman, but also when he begins his family life with an uncertain reality. Adaptations in married life, i.e., being between love and pain, hope and achievement, failure, and then bouncing back [68], lead to a successful family, encouraging other family members to remain in synergy, and as human development occurs continuously. Adaptation is the adjustment of family members to the process of life.

### 3.3 Family adaptation based on gender, environment, and profession

The parenting pattern of a mother, the relationship between mother and child, and the rules made by the mother for her children are said to be the best predictors of family adaptation [69]. The family is a system that can adapt to new situations with various influences from stimuli, and the adaptation mechanism depends on their coping abilities [70]. Adaptation is defined as the ability to adapt to changes in the environment, in which the family and environment mutually influence the adaptation process. Changes in priorities within the family, i.e., the situation the family faces and their learning from it are part of an adaptation in which the family needs to manage resources and develop the family potential (Nadrowska, Błażek, and Lewandowska-Walter, 2021) [[71], [72]].

Women who are successful in adapting when they become part of the family institution and build their own small families will be able to understand their roles and duties at every stage and process, thus achieving family harmony easily. Another study shows that in terms of family function, women who are married and understand their role and function in the family will have better adaptation skills (Kim, Kim, and Joh, 2015). When the function of the family becomes a strong root in living a married life, it reduces the psychological impact of the ups and downs of life in the marriage process. Conversely, if a married woman with low adaptive power is unable to understand the functioning of the family, she is more likely to be psychosocially disturbed, which reduces her subjective well-being.

Factors affecting the adaptation and resilience of African-American women with low incomes in urban areas indicated that adaptive capacity was implemented to adjust to the situation of family life by examining vulnerability, resilience, and demographics. The ability of a family to adapt to life situations is influenced by partnerships, growth, affection, and determination to remain resilient. Urban mothers adapt by teaching their moral principles and belief in goodness to ensure that their children make better decisions in the future and achieve their potential and life goals. Children born in this low economic line are protected from the risks of financial danger in their lives as experienced by their families [73].

Humans experience two things when making adaptations that mediate adaptation in the form of social support: the ability of a family to deal with urgent situations, and spiritual beliefs [74]. Simultaneously, the barriers to adaptation are in the form of mental stress, chronic illness, and other conditions that are less favorable to the family institution. Humans are experiencing the COVID-19 outbreak; therefore, family institutions that can adapt will awaken the family facing the test [59,75]. Adaptations by farming families in rural areas can form ways by which these families can deal with climate change, such as the local environment, temperature, extreme rainfall, stock availability, and production relativity, which forces them to meet harvest goals and face changes in ecology and the environment (Socioeconomic systems) (Mihiretu, Okoyo, and Lemma, 2019; Aslany and Brincat, 2021; Destaw and Fenta, 2021).

Rural families with jobs in agriculture are the key to ensuring their food security and sustainable development. Adaptations in farming families are related to the mitigation of the physical environment, such as reducing emissions from deforestation and greenhouse gases, as well as reducing waste [76]. The results of other studies also show that age, education, number of family members, land area owned, seed prices, and ease of accessing agricultural technology are determinants of adaptive capacity in farming families (Aniah, Kaunza-Nu-Dem, and Ayembilla, 2019; Maya, K. A., Sarker, M. A. R., & Gow, 2019; Naqvi et al., 2020). In farming families, adaptation is largely influenced by the decisions of the head of the household (husband or father), family size, access to extension services, size of owned land, frequency of experiencing crises in farming (Amare and Simane, 2018; Al-Amin et al., 2019; Muroyiwa, Masinda, and Mushunje, 2021), social capital [77], agroecology, and farming experience [78].

The adaptation of rural families working as breeders is influenced by family characteristics in the form of the roles of family members according to marital status [79], genders, such as differences in the roles of fathers and mothers as breeders, age, education level, number of family members, experience raising livestock, income in animal husbandry, non-livestock income, land area owned, number of livestock, size of cattle pens, access to information, access to capital to develop livestock, access to training by extension workers, and perceptions of farmers in increasing livestock productivity (Mihiretu, Okoyo, and Lemma, 2019).

Adaptation to the family environment in rural areas aims to balance economic, social, and household environments [80]. Rural families were also found to be positively adaptable by being influenced by the way the head of the family, namely the father or husband, makes decisions [81]. Adaptation is an effective human response to changes in natural systems that encourages the human ability to understand opportunities and challenges, reduce harm, and profitably take advantage of opportunities [45].

Similar to farming families, fishing families in rural areas also respond to adaptation as a strategy to deal with the various pressures and risks of climate change, limited resources, capital ownership, and access. Simultaneously, in the internal family, the adaptation of fishermen to working hours, stress pressure experienced due to responsibilities in their roles as parents and partners to maintain conditions from economic decline play a role in their adaptation [82].

Adaptation differs between married men and women. Predictive factors in the adaptation of a father or husband are marital satisfaction, the ability to perform the role of a father, stability in a career, and property owned to care for and carry out obligations as the head of the household [83]. The adaptations carried out by women is driven more by internalization problems that exist within themselves than by the profile of external problems [84].

For example, the model of adaptation according to professions in a community culture with academic partners who have careers at universities shows that the birth of a child forces parents to adapt to each other through new life scenarios, greater responsibilities, boundaries, and opportunities [85]. Typically, dedicating oneself as an academician by working at a university has a fairly high level of work flexibility compared to that of a family with other professional partners. However, when one has children, their focus and concentration split to carry out academic obligations, making it quite difficult to adjust to their needs and childcare, particularly when a child requires more attention. As children grow and mature, adaptation has a major impact on their lives and career advancement, making it easy for parents to deal with unexpected family problems [86]. The adaptation of married couples who work on the investment in time and capital to understand each other would create a more prosperous family environment.

Families that need greater adaptation are characterized by low-income families with risk factors for social inequality, economic difficulties, and binding sociocultural norms [87]. The environment as a system in family adaptation supports adaptation strategies, including (a) proactive involvement of every family member, (b) equality and responsibility, (c) nature-based approaches, and (d) mainstreaming of systematic adaptation involving regional governance and environmental management of impact risks [88].

### 3.4 Family adaptation based on coping

Research results from a systematic review conducted by earlier researchers, similar to that in this study using PRISMA to evaluate the mental health of family members and children during the COVID-19 pandemic from 17 articles, stated that during the process of social isolation, individuals in family institutions felt anxiety, stress, and fear. The worst potential for mental health and a higher family burden during a pandemic are shown by rural families, women, and the older adults. To build family coping strategies so that each family member can adapt during the COVID-19 pandemic, a familyfriendly policy with flexibility is required (Fong & Larocci, 2020).

Anxiety during COVID-19 experienced by families causes insomnia, heteroaggression, verbal or physical abuse in children, increased alcohol consumption, drug abuse, aggressiveness, mood swings, and suicidal ideation. One factor that can help avoid these risks is improving stress management and family coping skills. Parents who have coping skills and can implement stress management strategies have healthier objective and subjective wellbeing, especially when dealing with COVID-19, which encourages health, economic, and social inconsistencies. At critical times, every family member must carry out psychological interventions to minimize depression, increase hope for the future, be ready for future challenges, and build a physically and mentally healthy community, starting with the family (Kandula and Wake, 2022).

Another survey conducted by previous researchers stated that during the COVID-19 pandemic, women used adaptive coping more often than men. The impact of the inability of an individual to manage stress and lack of skill in coping is the behavior of denying, blaming oneself, and using substances or illegal drugs, which triggers psychopathology. This study provides suggestions for overcoming coping problems, namely self-acceptance, positive thinking, continuing to hone coping independently, understanding the disturbances felt by oneself, careful planning to achieve life goals, religion, and if you need emotional help then you need to seek help, nternal and professional. Adaptive coping is performed more often by individuals when facing life challenges than maladaptive coping. Not engaging in maladaptive coping helps individuals adapt better. The coping abilities of a person and adaptation to solving life problems generally vary and are determined by demographics, experience, and planning (Singh, Patra, & Singal, 2021).

## 4 Discussion and future research

The early months of 2020 marked the outbreak of the coronavirus, which dominated and changed the lives of everyone globally. Life changed instantly with learning from home, distancing from home, and working from home. As every rule has consequences, the new regulations passed by governments in some parts of the world created social uncertainty and fear [89]. Fear is the emotion that arises when a human faces a real threat or perceives an obstacle. Some physiological symptoms, such as heart palpitations, stiff muscles, an increase in pulse rate, and other psychological symptoms in the form of a response to anger, avoidance, screaming, or even silence (Siddique, Ahmed, and Hossain, 2021).

No one knew when the pandemic would end; therefore, humans faced challenges in economic shocks and changes in social behavior [90]. Currently, family adaptation required in a global context is a way for families to survive the COVID-19 pandemic. In the largest COVID-19-affected country globally, family adaptation significantly increases solidarity networks, strengthens relationships between family members both internally and externally, develops new technologies in family domestic work, opens communication relationships, and balances work patterns and family harmony [91] due to the increased flexibility of work during COVID-19 by working from home.

Adaptation to the family environment involves all family members and various emotional and physical components. Ecological and psychosocial studies state that the COVID-19 pandemic has led to a decline in the sense of social ownership in the community that changes the original human function as a social species, which always communicates with other people as a basic fulfillment in daily life, has now become separated and full of limitations. Thus, the inability of the family environment to become a place to learn to adapt to new conditions will cause individuals to have a social identity crisis (Antonini Philippe, Schiavio, and Biasutti, 2020). Limiting physical, psychological, emotional, and social distancing from the world outside the home environment would lead to negative emotional experiences. With effective communication, mutual support, and care for the physical and mental health of each other, each family member can build good adaptation.

The adaptation strategy is not obtained by the family from birth or is only referred to as the obligation of a husband or father to protect their family. Adaptation strategies need to be developed and is a way of overcoming problems by the family in meeting life needs, which is called a coping strategy [92]. Education, income, and household perceptions have a positive effect on household adaptation (Gebru, Ichoku, and Phil-Eze, 2020). Gender also affects adaptability, where men as the head of the family are believed to have a higher adaptive capacity than women [93].

When humans are required to adapt to new ways of living and working, the family is the main factor in their survival and determines the extent to which individuals can adapt to COVID-19; thus, the family environment can be stated as a key factor in adapting to new habits during COVID-19. The pandemic has also changed many arrangements, such that eventually humans do not need to wait for the outbreak to end, and the adaptation in the family environment ensures that families can survive even though they have to coexist with COVID-19. With COVID-19, humans can be interpreted using the theory of social learning and planned behavior. The existence of these events considers family background the presence of social support, and the influence of the role model on dominant family behavior [94].

After analyzing scientific publications and extracting them as a model of adaptation to the family environment in the face of the COVID-19 pandemic, although the number of academic publications found has increased significantly, especially in discussions of scientific adaptation, such as biology, medicine, and health, the phenomenon of adaptation in the family environment specifically show the form of adaptation during the COVID-19 pandemic in the social science field. However, further development is required. Conclusively, the increasingly rapid changes in global environmental capacity that are increasingly unpredictable during COVID-19 can be a special field that can be researched according to other scientific research fields.

## 5 Limitation and conclusion

This study has some limitations. The data collection method conducted using a literature review has the potential to be analyzed extensively and may result in an analysis bias towards correlated phenomena. Furthermore, few researchers have examined the adaptation to the family environment in the context of COVID-19, thus limiting the search for relevant studies.

The research data state that adaptation to the family environment can be developed based on the grand theory: (a) Social Science, (b) Ecology, (c) Cultural Science, (d) Economics, and (d) Psychosocial Science. Family adaptability can be measured through (1) acceptance of family members in dealing with life, (2) management of available resources, (3) ability to see opportunities, and (4) commitment to provide social, emotional, and physical support to each family member.

The model or pattern of family adaptation found in this study stems from situational events in the family environment, and to adapt and solve the problem, the family needs to consider family culture and values. Generally, family adaptation occurs due to stress, and each family member adopt a coping process through social support interactions and transactions to help families achieve family welfare both objectively and subjectively.

Adaptation to the family environment during the COVID-19 pandemic; (a) is a life process required by family institutions to maintain their ability to balance their roles and functions, (b) the COVID-19 pandemic, which has changed the global system and environment, has created pressure, especially stress (emotional and financial) and depression, (c) adaptation is required for the life events that trigger this imbalance, especially in the family environment. The ability of family members to adapt will create happiness and resilience in the face of the critical situation and motivate every family member, (d) the COVID-19 outbreak can be regarded as a case study to learn and understand the processes of family adaptations.

## Author contribution statement

All authors listed have significantly contributed to the development and the writing of this article.

## Data availability statement

Data included in article/supplementary material/referenced in article.

## Funding statement

This research was a part of the Higher Education Excellence Research Grant, 10.13039/501100016270Jakarta State University, for the 2021 fiscal year in the field of Environmental Education. The research team would like to thank the Ministry of Education, Culture, Research, and Technology of the Republic of Indonesia (Kemendikbud-Ristek RI), Institute for Research and Community Service (LPPM), and Faculty of Engineering at Jakarta State University.

## Declaration of competing interest

The authors declare that they have no known competing financial interests or personal relationships that could have appeared to influence the work reported in this paper.
