# Supplementary material for: Understanding adaptability in the family environment in facing COVID-19: A review
Source: Heliyon. 2023 Oct 27;9(11):e20618. doi: 10.1016/j.heliyon.2023.e20618 (PMC10665684; doi:10.1016/j.heliyon.2023.e20618)
Supplement: Multimedia component 1 [file mmc1.pdf]

PRISMA 2020 Checklist

Section and Topic	Item #	Checklist item	Location where item is reported
**TITLE**			
Title	1	Identify the report as a systematic review.	
**ABSTRACT**			
Abstract	2	See the PRISMA 2020 for Abstracts checklist.	
**INTRODUCTION**			
Rationale	3	Describe the rationale for the review in the context of existing knowledge.	
Objectives	4	Provide an explicit statement of the objective(s) or question(s) the review addresses.	
**METHODS**			
Eligibility criteria	5	Specify the inclusion and exclusion criteria for the review and how studies were grouped for the syntheses. in page 3	v
Information sources	6	Specify all databases, registers, websites, organisations. reference lists and other sources searched or consulted to identify studies. Specify the date when each source was last searched or consulted.	
Search strategy	7	Present the full search strategies for all databases, registers and websites, including any filters and limits used.	
Selection process	8	Specify the methods used to decide whether a study met the inclusion criteria of the review, including how many reviewers screened each record and each report retrieved, whether they worked independently, and if applicable, details of automation tools used in the process.	
Data collection process	9	Specify the methods used to collect data from reports, including how many reviewers collected data from each report, whether they worked independently, any processes for obtaining or confirming data from study investigators, and if applicable, details of automation tools used in the process.	
Data items	10a	List and define all outcomes for which data were sought. Specify whether all results that were compatible with each outcome domain in each study were sought (e.g. for all measures, time points, analyses), and if not, the methods used to decide which results to collect.	
	10b	List and define all other variables for which data were sought (e.g. participant and intervention characteristics, funding sources). Describe any assumptions made about any missing or unclear information.	
Study risk of bias assessment	11	Specify the methods used to assess risk of bias in the included studies, including details of the tool(s) used, how many reviewers assessed each study and whether they worked independently, and if applicable, details of automation tools used in the process.	
Effect measures	12	Specify for each outcome the effect measure(s) (e.g. risk ratio, mean difference) used in the synthesis or presentation of results.	
Synthesis methods	13a	Describe the processes used to decide which studies were eligible for each synthesis (e.g. tabulating the study intervention characteristics and comparing against the planned groups for each synthesis (item #5)).	
	13b	Describe any methods required to prepare the data for presentation or synthesis, such as handling of missing summary statistics, or data conversions.	
	13c	Describe any methods used to tabulate or visually display results of individual studies and syntheses.	
	13d	Describe any methods used to synthesize results and provide a rationale for the choice(s). If meta-analysis was performed, describe the model(s), method(s) to identify the presence and extent of statistical heterogeneity, and software package(s) used.	
	13e	Describe any methods used to explore possible causes of heterogeneity among study results (e.g. subgroup analysis, meta-regression).	
	13f	Describe any sensitivity analyses conducted to assess robustness of the synthesized results.	
Reporting bias assessment	14	Describe any methods used to assess risk of bias due to missing results in a synthesis (arising from reporting biases).	
Certainty assessment	15	Describe any methods used to assess certainty (or confidence) in the body of evidence for an outcome.	

PRISMA 2020 Checklist

Section and Topic	Item #	Checklist item	Location where item is reported
**RESULTS**			
Study selection	16a	Describe the results of the search and selection process, from the number of records identified in the search to the number of studies included in the review, ideally using a flow diagram. in page 5	v
	16b	Cite studies that might appear to meet the inclusion criteria, but which were excluded, and explain why they were excluded. in page 5	v
Study characteristics	17	Cite each included study and present its characteristics.	
Risk of bias in studies	18	Present assessments of risk of bias for each included study.	
Results of individual studies	19	For all outcomes, present, for each study: (a) summary statistics for each group (where appropriate) and (b) an effect estimate and its precision (e.g. confidence/credible interval), ideally using structured tables or plots.	
Results of syntheses	20a	For each synthesis, briefly summarise the characteristics and risk of bias among contributing studies.	
	20b	Present results of all statistical syntheses conducted. If meta-analysis was done, present for each the summary estimate and its precision (e.g. confidence/credible interval) and measures of statistical heterogeneity. If comparing groups, describe the direction of the effect.	
	20c	Present results of all investigations of possible causes of heterogeneity among study results.	
	20d	Present results of all sensitivity analyses conducted to assess the robustness of the synthesized results.	
Reporting biases	21	Present assessments of risk of bias due to missing results (arising from reporting biases) for each synthesis assessed.	
Certainty of evidence	22	Present assessments of certainty (or confidence) in the body of evidence for each outcome assessed.	
**DISCUSSION**			
Discussion	23a	Provide a general interpretation of the results in the context of other evidence. in page 5	v
	23b	Discuss any limitations of the evidence included in the review. in page 5	v
	23c	Discuss any limitations of the review processes used. in page 5	v
	23d	Discuss implications of the results for practice, policy, and future research. in page 5	v
**OTHER INFORMATION**			
Registration and protocol	24a	Provide registration information for the review, including register name and registration number, or state that the review was not registered.	
	24b	Indicate where the review protocol can be accessed, or state that a protocol was not prepared.	
	24c	Describe and explain any amendments to information provided at registration or in the protocol.	
Support	25	Describe sources of financial or non-financial support for the review, and the role of the funders or sponsors in the review.	
Competing interests	26	Declare any competing interests of review authors.	
Availability of data, code and other materials	27	Report which of the following are publicly available and where they can be found: template data collection forms; data extracted from included studies; data used for all analyses; analytic code; any other materials used in the review.	

From: Page MJ, McKenzie JE, Bossuyt PM, Boutron I, Hoffmann TC, Mulrow CD, et al. The PRISMA 2020 statement: an updated guideline for reporting systematic reviews. BMJ 2021;372:n71. doi: 10.1136/bmj.n71

PRISMA Table Number 5

The research sample provisions that we carry out are subject to the following conditions or criteria:

a. Inclusion criteria

Inclusion criteria are subjective characteristics generally taken from journals or generic articles covering the research topic. The inclusion criteria in this study were family ecology and family adaptation.

b. Exclusion criteria

Exclusion criteria are subjects who meet the inclusion criteria in accordance with the research objectives. The inclusion criteria in this systematic literature review article are families facing the COVID-19 virus outbreak and must adapt because of this world problem.

PRISMA Table Number 16

We explain table 16a in figure 2 as follows:

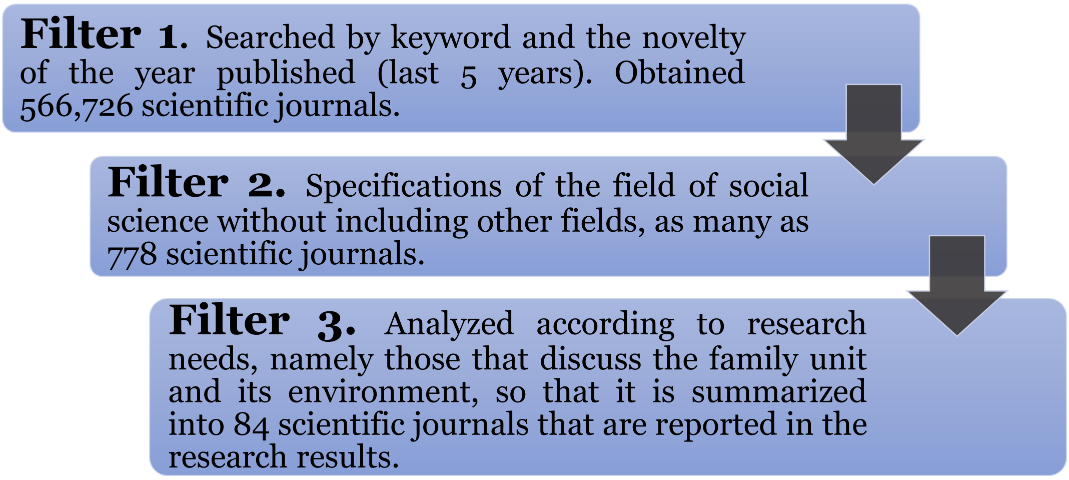

Fig. 2 - Flow chart representing the process of filtering literature review source data. Source: Research data analysis (2021)

We describe table 16b in figure 3 below:

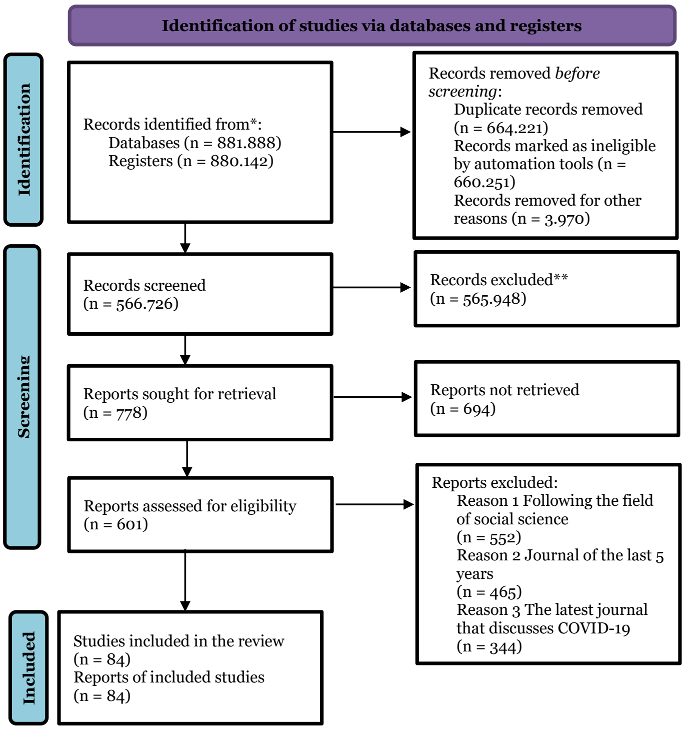

* If feasible, we reported the number of records identified from each database or register searched (rather than the total number across all databases/registers).
**If automation tools were used, we indicated how many records were excluded by a human and how many were excluded by automation tools.

Fig. 3 - PRISMA diagram depicting the process of the systematic literature review. Source: Research data analysis (2021)

PRISMA Table Number 23

We present table 23 in the following context:

See page 6

The topic of adaptation was discussed in 206,117 journal articles. In this study, the topic was adapted to the research objective, namely, to see adaptation in the family environment. 67,377 journal articles appeared in the search for scientific journals. We identified 23 articles that were suitable for further research. In the family adaptation variable, we generally looked for a family coping with challenges that trigger stress, which we re-examined in a systematic literature review of 10 journal articles. Globally, families are trying to deal with epidemics and disasters, which result in uncertainty in life. Families require adaptation to survive negative impacts. For more than a year, COVID-19 has become an issue that has resulted in instability of life. Several changes have occurred a rapid pace, and humans must adjust to these changes quickly.

See page 8

Table 1. Family adaptation system. By researcher (2021)

Family Adaptation System		Life Cycle	Aim	Process
1.	Parenting pattern	Children to adults	Parenting shapes the adaptability of children, so that when children can adapt, it will be easy to accept adaptation in the adult phase.	Make rules, form bonds, and build relationships between parents and children
2.	Environmental change	Long-term and short-term in individual	Drive change to occur within the individual strongly based on experience and environmental changes.	Anticipate and train someone to have adaptation strategies, discover potential, and develop their resources.
3.	Coping	When one of the family members is sick, separated from the nuclear family and social environment, and experiencing changes in routine	Adjusting to differences, confusion, and difficulties, reducing negative prejudices, the ability to face obstacles, and giving affirmation that individuals as family members who are going through a difficult phase of life still have strength and help from other family members	Opening positive communication, creating interactions that can reduce anger and despair, as well as family transactions with the social environment
4.	Family welfare	Availability of resources, conditions in which family members feel	Adapting to life situations	Understanding risks and dangers, instilling mutual respect among family members, receiving and responding to love and

		satisfied, fulfilled, happy, and have gratitude		affection, respecting human nature, and exchanging needs for mutual fulfillment
5.	Emotional Response and regulation	Children to teenagers	Responses were given by family members, understanding of others, developing the capacity to understand and accommodate the needs and feelings of others	Accurate description of expressions and response cues, communication patterns as an adaptation style, and individuals as family members can be accepted by their environment and accept their environment
6.	Acculturation, assimilation, cultural processes, and family life history	Human life	Live life wisely, and understand that the essence of life is to change	Marrying different ethnic groups, having life events beyond human control, changing places of residence, getting new cultures and habits, meeting new people, and being part of a society with certain customs.

See page 11

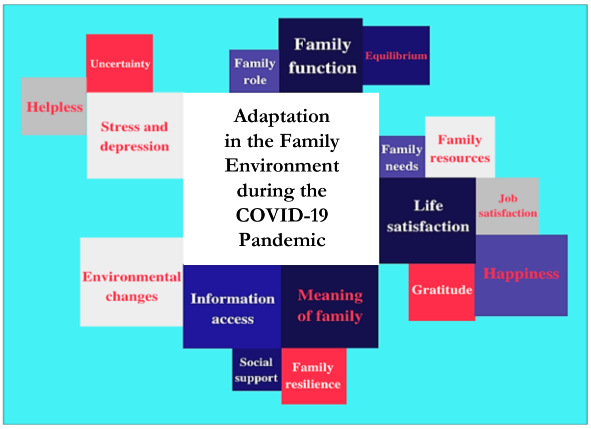

Fig. 5 - Chart showcasing the understanding of adaptation in the family environment in the COVID-19 pandemic situation. Source: Research data analysis (2021)

See page 18

The model or pattern of family adaptation found in this study stems from situational events in the family environment, and to adapt and solve the problem, the family needs to consider family culture and values. Generally, family adaptation occurs due to stress, and each family member adopt a coping process through social support interactions and transactions to help families achieve family welfare both objectively and subjectively.

Adaptation to the family environment during the COVID-19 pandemic; (a) is a life process required by family institutions to maintain their ability to balance their roles and functions, (b) the COVID-19 pandemic, which has changed the global system and environment, has created pressure, especially stress (emotional and financial) and depression, (c) adaptation is required for the life events that trigger this imbalance, especially in the family environment. The ability of family members to adapt will create happiness and resilience in the face of the critical situation and motivate every family member, (d) the COVID-19 outbreak can be regarded as a case study to learn and understand the processes of family adaptations.
